# Associação de Fatores de Risco Cardiovascular e Polimorfismo de APOE com Mortalidade em Idosos Longevos: Uma Coorte de 21 Anos

**DOI:** 10.36660/abc.20190263

**Published:** 2020-11-01

**Authors:** Lilian Vivian, Neide Maria Bruscato, Berenice Maria Werle, Waldemar de Carli, Renata Alonso Gadi Soares, Paulo Caleb de Lima Santos, Emilio Hideyuki Moriguchi

**Affiliations:** 1 Hospital Comunitário São Peregrino Lazziozi Associação Veranense de Assistência em Saúde VeranópolisRS Brasil Hospital Comunitário São Peregrino Lazziozi, Associação Veranense de Assistência em Saúde (AVAES), Veranópolis, RS - Brasil; 2 Hospital das Clínicas da Universidade de São Paulo Instituto do Coração (InCor) Laboratório de Genética e Cardiologia Molecular São PauloSP Brasil Laboratório de Genética e Cardiologia Molecular, Instituto do Coração (InCor), Hospital das Clínicas da Universidade de São Paulo (HC/FMUSP), São Paulo, SP - Brasil; 3 Universidade Federal de São Paulo Escola Paulista de Medicina Departamento de Farmacologia São PauloSP Brasil Universidade Federal de São Paulo Escola Paulista de Medicina - Departamento de Farmacologia, São Paulo, SP - Brasil; 4 Universidade Federal do Rio Grande do Sul Medicina Interna Porto AlegreRS Brasil Universidade Federal do Rio Grande do Sul - Medicina Interna, Porto Alegre, RS- Brasil

**Keywords:** Doenças Cardiovasculares, Fatores de Risco, Mortalidade, Apoliproteina E4, Idoso de 80 Anos ou mais

## Abstract

**Fundamento:**

O conhecimento dos fatores ambientais e genéticos para um envelhecimento bem-sucedido em idosos longevos é controverso. Acrescenta-se a esta evidência, o fato de serem poucos os estudos delineados com essa população.

**Objetivo:**

Investigar a relação entre os genótipos mais frequentes da apolipoproteína E (APOE) e a mortalidade em idosos longevos que vivem em comunidade e sua sobrevida de acordo com os fatores de risco cardiovascular.

**Métodos:**

Uma amostra de 74 idosos com 80 anos ou mais da coorte do Projeto Veranópolis foi selecionada para genotipagem da APOE. Na linha de base, foram coletadas variáveis antropométricas, dosagens sanguíneas de glicose e lipídeos, pressão arterial e variáveis de estilo de vida (tabagismo, consumo de álcool e atividade física). A escala Bayer de Atividades da Vida Diária foi aplicada aos cuidadores dos idosos. O tempo de seguimento total do estudo foi 21 anos. Um p<0,05 bicaudal foi considerado estatisticamente significativo.

**Resultados:**

Não encontramos associação entre os genótipos da APOE e mortalidade. Entretanto, o risco de morte em idosos fumantes foi 2,30 vezes (*hazard ratio* [HR]; intervalo de confiança de 95% [IC 95%] 1,01 a 5,24); em diabéticos, 3,95 vezes (HR; IC 95% 1,27 a 12,30) do risco dos não diabéticos. Indivíduos que praticavam atividade física vigorosa tiveram uma redução no risco de óbito em 51% (HR = 0,49; IC 95% 0,27 a 0,88). Para o aumento de 1 mmHg na pressão arterial sistólica houve uma redução de 2% (HR = 0,98; IC 95% 0,97 a 0,99) no risco de morte.

**Conclusão:**

Nesta amostra de longevos, não houve associação entre os genótipos da APOE e mortalidade. Entretanto, os fatores de risco cardiovasculares clássicos podem ser importantes para a mortalidade geral em pessoas muito idosas.

## Introdução

O rápido crescimento da população idosa em todo o mundo desperta o interesse e a necessidade de estudos sobre os fatores para alcançar uma longevidade com qualidade de vida. Dados de mortalidade em idosos com mais de 80 anos mostram que as doenças cardiovasculares (DCV) representam metade das causas de óbito.^1^ Apesar da frequência de doenças crônicas, como as cardiovasculares ou demências, aumentarem com a idade, idosos acima dos 80 anos, em geral, são excluídos dos estudos bem controlados ou são analisados como subgrupos. Quando incluídos, surgiram resultados diferentes dos encontrados para idosos jovens (60 a 74 anos), como maior mortalidade associada à diminuição da pressão diastólica^2^ ou da pressão sistólica^3^^,^^4^ e à redução do colesterol,^5^ ou ainda, um efeito protetor relacionado a um índice de massa corporal (IMC) acima de 30 kg/m^2^. ^6^ No entanto, outros fatores de risco como tabagismo^7^ e diabetes mellitus (DM)^8^ tiveram associação semelhante mesmo em idades mais avançadas. De outra forma, um fator genético extensamente estudado, o polimorfismo da apolipoproteína E (APOE), mais especificamente o alelo ε4, mostrou-se como fator de risco para demência de Alzheimer em adultos e idosos jovens.^9^^,^^10^ Entretanto, resultados de uma coorte específica de idosos longevos identificaram um efeito paradoxal do alelo ε2 da APOE associado a um aumento da doença de Alzheimer (DA) por critérios neuropatólogicos *post mortem*.^11^ Estudos de metanálises mostraram que os portadores do alelo ε4 da APOE apresentam maior risco de DCV.^12^^,^^13^ No entanto, não existem estudos que indiquem se esta associação se mantém na faixa etária dos longevos. Por este panorama, o objetivo deste estudo é investigar a relação entre os genótipos mais frequentes da APOE e a mortalidade em indivíduos longevos e descrever a sobrevida conforme os genótipos e a exposição a fatores de risco cardiovascular clássicos.

## Métodos

### Delineamento

Estudo de coorte prospectivo

### População Investigada

A coorte do Projeto Veranópolis iniciou em 1994 com dois critérios abrangentes de elegibilidade: (1) idade igual ou superior a 80 anos e (2) residência no domínio territorial do município de Veranópolis, RS, Brasil. O recrutamento dos idosos elegíveis ocorreu em 1994, 1996 e 1998. Brevemente, o primeiro recrutamento aconteceu durante o mês de julho de 1994 através de um convite informal do coordenador da pesquisa aos participantes de uma cerimônia religiosa. Espontaneamente, os presentes inscreveram-se e comunicaram aos seus próximos. Durante três semanas do mês de julho de 1994, os pesquisadores visitaram 100 idosos em suas próprias residências ou em centros comunitários. Em 1996, com uma estrutura formalizada e por divulgação em emissora de rádio local, mais 129 idosos consentiram em participar do estudo e os que já haviam participado em 1994 foram reavaliados. Em 1998, uma amostra aleatória simples dos participantes dos anos anteriores e mais 13 voluntários novos fizeram a genotipagem da APOE e os principais testes das avaliações pregressas. Assim, 242 indivíduos constituíram a coorte do Projeto Veranópolis. Número que perfez 87,4% dos idosos com 80 anos ou mais residentes no município entre os anos de 1994 e 1998.^14^

Durante 2011 e 2012, o *status* vital dos idosos amostrados para a genotipagem da APOE (74 indivíduos) foi verificado, através de visitas domiciliares e, nesta oportunidade, também, aplicou-se o questionário da Escala Bayer de Atividades da Vida Diária (B-ADL) aos cuidadores dos idosos.

Em dezembro de 2012, 18 anos após o início da coorte, haviam 11 longevos vivos de um total de 242 idosos. Os resultados apresentados neste manuscrito referem-se a um período após a verificação do *status* vital de 2011 a 2012, ou seja, após a ocorrência do desfecho mortalidade de todos os participantes da coorte de longevos, ocorrido em 2015.

O estudo foi aprovado pelo Comitê de Ética em Pesquisa da Universidade Federal do Rio Grande do Sul. Todos os participantes e/ou seus familiares assinaram o termo de consentimento livre e esclarecido.

### Variáveis

Utilizaram-se nesta avaliação da coorte de Veranópolis os genótipos da APOE (rs7412 e rs429358) como variável preditora, um fator genético analisado em dois momentos durante o seguimento: (1°) em 1998^15^ e (2°) em 2011, todos os indivíduos vivos que não haviam sido amostrados em 1998, ou seja, mais 9 idosos (metodologia descrita em Alvim et al.^16^ (Ver Anexo Fluxograma)

O desfecho definido neste estudo foi mortalidade por DCV crônica incluída nos códigos I00-I99 ou por demência incluída nos códigos F00-F03 da décima revisão da Classificação Estatística Internacional de Doenças e Problemas Relacionados à Saúde (CID-10). Para a definição da causa *mortis* do indivíduo idoso, foram apresentadas as segundas vias das certidões de óbito a dois profissionais médicos, um geriatra e outro cardiologista, que estiveram cegados entre si e com relação aos genótipos dos falecidos. Nos casos de divergência entre os profissionais com relação à causa do óbito, foi solicitada a avaliação por um terceiro profissional. O diagnóstico final, em caso de impasse, foi definido por consenso dos três profissionais. Quando não foi possível definir a causa de morte apenas pelo documento, foram levantados dados de prontuários e entrevistas com os médicos das famílias ou com os familiares do idoso falecido.

A coorte do Projeto Veranópolis é um estudo amplo de variáveis que busca respostas para a longevidade peculiar desta população. Dentre as diversas variáveis investigadas na coorte, selecionou-se para este estudo aquelas que são descritas como fatores de risco clássicos para as DCV e, que poderiam estar associadas de forma independente ao desfecho estudado: hipertensão arterial, obesidade, DM, dislipidemia, tabagismo, abuso do álcool e inatividade física. Dados destas variáveis foram coletados na linha de base do ano de inclusão do idoso na coorte (1994, 1996 ou 1998). Os dados basais coletados em 1994 foram reavaliados em 1996. Em 1998, repetiu-se a coleta dos dados de uma amostra aleatória de idosos dos anos de 1994 e 1996 e mais 13 indivíduos incluídos na coorte. Na análise dos dados deste estudo utilizou-se as informações coletadas no ano da entrada do idoso na coorte.

Sucintamente, os métodos utilizados na mensuração dos fatores de risco cardiovasculares e as justificativas da categorização, quando aplicáveis, foram descritos a seguir. A pressão arterial (PA) foi obtida utilizando-se de um esfignomanômetro de mercúrio (Erka, Germany). Foram tomadas duas ou três medidas, conforme a variabilidade, aguardando-se o intervalo recomendado pelas diretrizes e fez-se a média ponderada. Para a análise dos dados, a PA foi utilizada como variável quantitativa e categorizada, sendo considerado o indivíduo hipertenso quando PA ≥ 140/90 mmHg ou em uso de medicamento anti-hipertensivo.^17^ Ainda, avaliou-se a pressão de pulso, resultado da subtração pressão arterial sistólica (PAS) − pressão arterial diastólica (PAD).

A obesidade foi definida pelo IMC, sendo o peso medido com os participantes minimamente vestidos, sem sapatos, utilizando-se de uma balança mecânica de contrapeso (Filizolla, São Paulo). A estatura foi determinada em pé, sem sapatos, utilizando-se de fita métrica, quando os ombros estavam em uma posição normal. Para a análise dos dados, o IMC foi utilizado como variável contínua e categórica, com definição de obesidade^18^ e excesso de peso^19^ pelos pontos de corte ≥ 30 kg/m^2^ (Organização Mundial da Saúde – OMS) e > 27 kg/m^2^ (Lipschitz), respectivamente.

Nas avaliações bioquímicas do perfil lipídico e da glicemia, foram coletadas amostras de sangue venoso, após 12h de jejum. Utilizou-se o sistema de venóclise com aparato descartável a vácuo (Vacutainer) em tubos sem anticoagulante. As dosagens plasmáticas foram realizadas através de técnica manual de reação enzimática colorimétrica com padrões de calibração e amostras em duplicata. DM foi definido como glicemia de jejum ≥ 126 mg/dL ou em uso de medicação hipoglicemiante.^20^ A dislipidemia foi avaliada através das dosagens plasmáticas de triglicerídeos (TG), colesterol total (CT), colesterol da lipoproteína de alta *densidade ou high density lipoprotein* (HDL-C) e do cálculo do colesterol da lipoproteína de baixa densidade ou *low density lipoprotein* (LDL-C), sendo utilizadas como variáveis quantitativas e categorizadas de acordo com os critérios da V Diretriz Brasileira de Dislipidemias e Prevenção da Aterosclerose^21^ e das Diretrizes da Associação Americana de Endocrinologia para Tratamento das Dislipidemias e Prevenção da Aterosclerose:^22^ TG ≥ 150 mg/dL; CT ≥ 200 mg/dL; HDL-C < 50 mg/dL para mulheres e < 40 mg/dL para homens; LDL-C ≥ 160 mg/dL. O LDL-C foi obtido através da fórmula de Friedwald, para valores de TG inferiores a 400 mg/dL.

As variáveis de estilo de vida - tabagismo, abuso de álcool e inatividade física - foram obtidas através de um questionário padronizado aplicado na linha de base. O tabagismo foi avaliado através do relato do consumo ou não de tabaco (cigarro, palheiro, cachimbo). Considerou-se dois grupos: 1) não tabagistas: indivíduos que nunca fumaram; 2) tabagistas ou ex-tabagistas. O abuso do álcool foi avaliado através do relato da quantidade de álcool ingerido por semana, sendo considerado uso abusivo valores > 210 g/semana para homens e > 105 g/semana para mulheres.^23^ A inatividade física foi avaliada através do relato das atividades diárias durante uma semana normal de trabalho e lazer. Utilizou-se dois diferentes pontos de corte: < 2.000 kcal/semana^24^^,^^25^ e < 4.000 kcal/semana,^26^ por serem quantidades mínimas de atividade física para alcançar benefícios cardiovasculares importantes como atenuação do espessamento da camada íntima-média das artérias carótidas,^24^ aumento do HDL-C^25^ e redução da mortalidade em pacientes com doença arterial coronariana.^26^ O instrumento utilizado para reportar as diferentes atividades físicas constituiu-se de uma lista de 27 atividades habituais da rotina de pessoas campesinas e citadinas e mais uma pergunta aberta sobre outra atividade além das pré-selecionadas. Solicitou-se aos participantes do estudo que reportassem o tempo gasto em minutos e a frequência semanal para desenvolver tais atividades. Seguindo o mesmo caminho de outros estudos de idosos longevos, optamos pela utilização do gasto energético calculado em quilocalorias por semana (kcal/sem) que considera o peso do participante, o tempo de atividade referido, os *metabolic equivalents* (MET) da atividade específica^27^ e a frequência semanal das atividades: Gasto energético (kcal/sem) = MET X Peso (Kg) X Tempo da atividade (minutos) / 60 X frequência semanal. Deste modo, julgamos que essa medida reflete melhor o gasto energético do idoso longevo da comunidade estudada (rural e urbana) do que a simples mensuração dos METs, usualmente descrita nos trabalhos atuais.

A B-ADL foi aplicada, por um pesquisador treinado e cegado com relação aos genótipos dos idosos, aos seus cuidadores no período de agosto de 2011 a dezembro de 2012. O instrumento foi empregado como forma de identificar casos de demência e o resultado utilizado como variável confundidora em potencial, uma vez que está bem descrito que o alelo ε4 da APOE é um fator de risco para o desenvolvimento da DA.^9^^,^^10^ O escore obtido com a B-ADL varia de 1,00 a 10,00 e representa maior dificuldade nas atividades da vida diária quanto maior a pontuação. Para a análise dos dados, o escore da B-ADL foi utilizado como variável quantitativa e categorizada, utilizando o ponto de corte ≥ 3,12 para definição dos casos de demência.^28^

### Análise Estatística

As variáveis quantitativas foram descritas por média e desvio padrão ou mediana e amplitude interquartílica. Para a comparação entre os grupos utilizou-se o teste t-Student para amostras independentes (teste de normalidade de Shapiro-Wilk) e, em caso de assimetria, o teste de Mann-Whitney. As variáveis qualitativas foram descritas através de frequências absolutas e relativas. Na comparação de proporções entre os grupos, o teste qui-quadrado de Pearson ou exato de Fisher foi aplicado. Utilizaram-se o método de estimação da curva por Kaplan-Meier para avaliar o tempo de sobrevida e o teste qui-quadrado de log-rank para a comparação entre os grupos.

Para controlar fatores de confusão em relação ao óbito, utilizou-se o modelo de azares proporcional de Cox. Como medida de efeito, foi calculada a razão de densidades de incidência (*hazard ratio* [HR]), com seus respectivos intervalos de 95% de confiança (IC 95%). O critério para a entrada da variável no modelo multivariado foi de que apresentasse um valor p < 0,20 na análise univariada.

O nível de significância adotado foi de 5% (p < 0,05) e os dados foram analisados com o programa *Statistical Package for the Social Sciences*, versão 21.0.

## Resultados

Neste trabalho, a amostra de 74 indivíduos da coorte de Veranópolis teve uma mediana de tempo de seguimento de 9 anos (P25 – P75: 6 – 14), com variação entre 0,6 e 21 anos. Salienta-se que não houve perdas de seguimento desta amostra. Com base no relato dos idosos, 94,6% eram descendentes de imigrantes italianos. A frequência gênica (alelos) da APOE presente na amostra foi: 4,1% ε2; 85,1% ε3 e 10,8% ε4. E a frequência genotípica: 1,4% E2E2; 5,4% E2E3; 71,6% E3E3 e 21,6% E3E4. A distribuição genotípica está em equilíbrio de Hardy-Weinberg (χ^2^ = 0,07; grau de liberdade = 1; p = 0,79). Não foram observados portadores dos genótipos E2E4 e E4E4 na amostra. Portanto, somente o genótipo E3E4 formou o grupo exposto, ou seja, portadores do alelo de risco ε4 da APOE. A Tabela 1 resume as características dos grupos de interesse. Esta tabela encontra-se completa, incluindo todas as variáveis descritas de forma quantitativa e categorizada, no Anexo 1: Tabela Completa 1.

**Tabela 1:** Caracterização da amostra

Variáveis	Amostra total (n = 69)	Genótipo E3E3 (n = 53)	Genótipo E3E4 (n = 16)	p*
Idade na entrada (anos)	82,6 ± 2,8	82,2 ± 2,7	84,0 ± 2,8	0,021
Sexo				0,267
Masculino	23 (33,3)	20 (37,7)	3 (18,8)	
Feminino	46 (66,7)	33 (62,3)	13 (81,3)	
B-ADL†	2,98 (1,44-5,55)	2,95 (1,38-5,46)	3,19 (1,54-7,44)	0,631
Tabagismo				0,717
Não fumante	56 (81,2)	42 (79,2)	14 (87,5)	
Fumante/Ex-fumante	13 (18,8)	11 (20,8)	2 (12,5)	
Uso abusivo de álcool				0,093
Não	17 (24,6)	13 (24,5)	4 (25,0)	
Sim	18 (26,1)	17 (32,1)	1 (6,3)	
Abstêmios	34 (49,3)	23 (43,4)	11 (68,8)	
IMC (Kg/m2)†	26,8 ± 5,4	27,6 ± 5,5	23,7 ± 3,6	0,011
Atividade física (kcal/semana)	5133 (2386-9846)	5421 (2155-10544)	3580 (2300-6930)	0,216
PAS (mmHg)	161 ± 25,3	162 ± 23,7	158 ± 30,5	0,489
PAD (mmHg)	86,3 ± 13,2	88,6 ± 12,7	78,5 ± 12,4	0,007
Pressão de pulso (mmHg)	75,1 ± 22,7	73,9 ± 21,5	79,0 ± 26,8	0,439
Hipertensão	62 (89,9)	48 (90,6)	14 (87,5)	0,660
Glicemia jejum (mg/dL)‡	95,4 ± 20,7	96,2 ± 22,7	92,6 ± 12,2	0,539
Colesterol total (mg/dL) ‡	209 ± 48,8	203 ± 43,9	229 ± 59,5	0,066
Colesterol LDL (mg/dL) ‡	138 ± 42,1	132 ± 36,0	156 ± 55,0	0,055
Colesterol HDL (mg/dL)§	43,4 ± 11,4	44,0 ± 12,5	41,4 ± 6,6	0,284
Triglicerídeos (mg/dL)‡	102 (85,8-146)	102 (83,4-144)	107 (88,2-159)	0,806

Resultados expressos por média ± desvio padrão, mediana (percentis 25–75) ou n (%).

* Teste t-student (comparação de médias), teste de Mann-Whitney (comparação de medianas), teste qui-quadrado de Pearson (variáveis categóricas) ou teste exato de Fisher (nas variáveis tabagismo e hipertensão);

† variáveis analisadas em 15 indivíduos do genótipo E3E4;

‡ variáveis analisadas em 52 indivíduos do genótipo E3E3;

§ variável analisada em 51 indivíduos do genótipo E3E3. B-ADL: Escala Bayer de Atividades da Vida Diária; IMC: índice de massa corporal; PAS: pressão arterial sistólica; PAD: pressão arterial diastólica; LDL: lipoproteína de baixa densidade; HDL: lipoproteína de alta densidade.

As causas de morte entre os grupos E3E3 e E3E4 estão resumidos na Tabela 2. Salienta-se que a expectativa de vida média para estes indivíduos foi de 92,3 anos (IC 95% 91,2 a 93,4). Para as comparações, na tabela, foram apresentados os níveis de significância sem ajuste e os ajustados às variáveis com p < 0,2 na análise univariada.

**Tabela 2:** Comparação dos desfechos entre os genótipos

Desfechos	Amostra total (n=69)	Genótipo E3E3 (n=53)	Genótipo E3E4 (n=16)	p	p^ajustado^‡
	n (%)	n (%)	n (%)		
Óbito	69 (100)	53 (100)	16 (100)	–	–
Causa do óbito				0,216*	0,302
Cardiovascular	43 (62,3)	36 (67,9)	7 (43,8)		
Demência	6 (8,7)	4 (7,5)	2 (12,5)		
Outras	20 (29,0)	13 (24,5)	7 (43,8)		

* Teste exato de Fisher;

‡ ajustado para idade na entrada do estudo, consumo de álcool, índice de massa corporal, atividade física ≥ 4000 kcal/semana, pressão arterial diastólica, colesterol total e colesterol da lipoproteína de baixa densidade.

**Figura 1:**
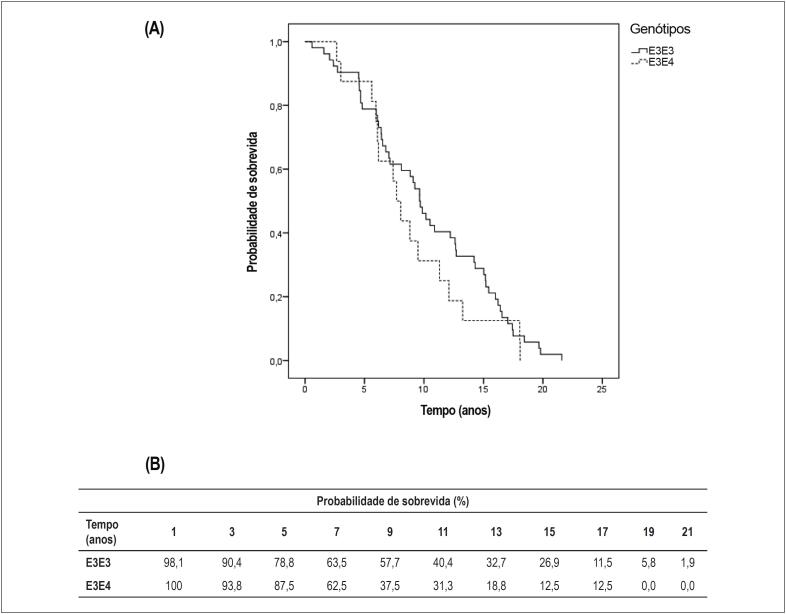
(A) Curva de sobrevida de Kaplan-Meier dos portadores dos genótipos E3E3 e E3E4. (B) Probabilidade de sobrevida para os grupos com periodicidade de 2 anos.

Para avaliar a sobrevida dos indivíduos idosos conforme os genótipos da APOE, utilizou-se a curva de sobrevivência estimada pelo método de Kaplan-Meier, representada no gráfico da Figura 1. Observamos que não houve associação entre os polimorfismos da APOE e sobrevida (logrank = 0,955) nos idosos longevos da amostra.

**Figura 2:**
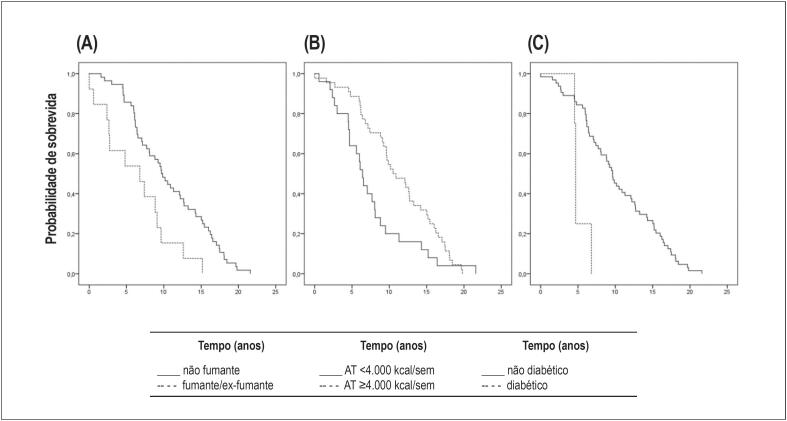
Curvas de sobrevida dos fatores associados à mortalidade em idosos longevos: (A) tabagismo, (B) atividade física [AT] vigorosa e (C) diabetes mellitus.

É pertinente analisar os fatores de risco cardiovasculares associados à mortalidade em idosos longevos, uma vez que esta faixa etária costuma apresentar resultados singulares e, ao mesmo tempo, contraditórios. Para isso, utilizou-se as estimativas de sobrevida de Kaplan-Meier com regressão de Cox para controlar confundimento. Os resultados estão apresentados na Tabela 3 (Anexo 2: Tabela Completa 3). As curvas de sobrevida dos fatores categóricos associados à mortalidade podem ser visualizadas na Figura 2.

**Tabela 3:** Regressão de Cox univariada e multivariada de fatores associados à mortalidade

Variáveis	Univariada	p	Multivariada*	p
	HR (IC 95%)		HR (IC 95%)	
Idade (anos) †	1,19 (1,10 – 1,30)	<0,001	1,24 (1,12 – 1,39)	<0,001
Sexo masculino	1,45 (0,88 – 2,39)	0,150	1,04 (0,47 – 2,32)	0,920
Genótipo E3E4	1,35 (0,77 – 2,39)	0,299	–	–
B-ADL	0,93 (0,85 – 1,02)	0,119	0,92 (0,82 – 1,02)	0,102
Fumante/Ex-fumante	2,37 (1,29 – 4,36)	0,005	2,30 (1,01 – 5,24)	0,047
Consumo de álcool (g/semana)	1,00 (0,99 – 1,00)	0,602	–	–
IMC	0,94 (0,90 – 0,99)	0,018	0,96 (0,91 – 1,01)	0,107
Atividade física ≥ 4000 kcal/sem	0,55 (0,34 – 0,89)	0,016	0,49 (0,27 – 0,88)	0,017
PAS	0,99 (0,98 – 1,00)	0,050	0,98 (0,97 – 0,99)	0,018
PAD	0,97 (0,94 – 0,99)	0,016	1,01 (0,98 – 1,04)	0,669
Pressão pulso	0,99 (0,98 – 1,01)	0,392	–	–
Hipertensão	0,53 (0,24 – 1,19)	0,123	1,35 (0,54 – 3,38)	0,516
Diabetes mellitus	5,10 (1,70 – 15,3)	0,004	3,95 (1,27 – 12,3)	0,018
Colesterol total ≥ 200 mg/dL	0,52 (0,32 – 0,86)	0,010	0,74 (0,42 – 1,31)	0,303
LDL ≥ 160 mg/dL	0,69 (0,41 – 1,18)	0,175	1,00 (0,99 – 1,00)	0,410
HDL baixo‡	1,46 (0,86 – 2,47)	0,159	1,03 (0,57 – 1,84)	0,932
Triglicerídeos ≥ 150 mg/dL	1,23 (0,69 – 2,20)	0,489	–	–

* O critério para a entrada da variável no modelo multivariado foi de que apresentasse um valor p < 0,20 na análise univariada.

† Idade do idoso no momento do recrutamento da coorte (entrada no estudo).

‡ HDL baixo refere-se a HDL-colesterol <40 mg/dL para homens e <50 mg/dL para mulheres. HR: hazard ratio; IC 95%: intervalo de 95% de confiança; B-ADL: Escala Bayer de Atividades da Vida Diária; IMC: índice de massa corporal; PAS: pressão arterial sistólica; PAD: pressão arterial diastólica; LDL: lipoproteína de baixa densidade; HDL: lipoproteína de alta densidade.

Considerando que a amostra deste estudo foi composta por idosos selecionados em dois momentos do seguimento, poderia ter ocorrido um viés de seleção. Especificamente, os nove idosos genotipados no ano de 2011 formariam um grupo de sobreviventes. Com o intuito de ponderar esse viés, refizemos as análises retirando estes indivíduos. Deste modo, obtivemos resultados bastante semelhantes, inclusive no que se refere ao nível descritivo amostral das variáveis, com exceção do tabagismo e diabetes que perderam a associação com mortalidade. Nesta nova análise, o risco de morte em indivíduos fumantes e ex-fumantes foi 2,14 (IC 95% 0,93 a 4,91) no modelo multivariado (p = 0,075).

## Discussão

### Polimorfismo da APOE

Estudos de revisão mostram que as frequências genéticas relacionadas ao polimorfismo da APOE são altamente variáveis, principalmente no que diz respeito ao alelo ε4.^29^ De modo geral, a frequência gênica observada no presente estudo foi semelhante à encontrada na população da Itália.^30^ Uma vez que a amostra deste trabalho continha 94,6% de descendentes de imigrantes italianos, essa similaridade era esperada e indica que o processo de amostragem foi adequado.

Curiosamente, nos nossos resultados, indivíduos com o genótipo E3E4 apresentaram uma idade média significativamente maior do que indivíduos E3E3 (Tabela 1). Acreditamos que esta diferença possa ser casual, uma vez que publicações relevantes indicam que não há diferença para mortalidade geral entre portadores dos genótipos E3E3 e E3E4 antes dos 80 anos.^31^^,^^32^ Dentre os demais fatores de risco para DCVs que foram investigados, o IMC e a PAD foram os que apresentaram diferenças significativas entre os genótipos avaliados. Nestes casos, indivíduos do grupo E3E3 apresentaram um IMC médio classificado como sobrepeso (OMS) ou excesso de peso (Lipschitz) e uma maior PAD. Não encontramos dados similares a estes em estudos previamente publicados de base comunitária, mas comparações semelhantes, em amostras maiores na faixa etária de adulto a idoso jovem, sugerem que não existe qualquer associação entre obesidade^33^ ou níveis de PAD^34^ e os genótipos da APOE.

### Fatores de Risco Cardiovascular Clássicos

Nossos resultados indicaram que alguns fatores de risco cardiovascular estão associados à mortalidade geral mesmo em idosos longevos, faixa etária na qual a maior parte destes fatores clássicos perde o seu poder preditivo de risco. O tabagismo mostrou ser importante nesta relação. O risco de morte em indivíduos fumantes e ex-fumantes foi 2,30 (IC 95% 1,01 a 5,24) vezes maior do que o risco em não fumantes. Uma metanálise recente^7^ torna evidente que o fumo permanece como um forte fator de risco para mortalidade prematura, também em idosos com mais de 80 anos. No que se refere aos idosos diabéticos, apesar do pequeno percentual presente na nossa amostra (6%), este número foi suficiente para alcançar uma diferença significativa. Os idosos longevos diabéticos tiveram 3,95 (IC 95% 1,27 a 12,3) vezes o risco de óbito dos não diabéticos. Resultado similar foi evidenciado no estudo da coorte longeva *The Adventist Health Study*.^8^ Outro resultado importante do nosso estudo, a atividade física vigorosa, apresentou-se como fator protetor para o óbito. Indivíduos que despendiam mais de 4.000 kcal/semana, em atividade de trabalho e lazer, tiveram uma redução no risco de óbito em 51% (IC 95% 12% a 73%). Com relação à prática de atividades vigorosas, um estudo que combinou duas coortes australianas, *Australian Longitudinal Study on Women's Health and the Health in Men Study*, com mais de 18 mil participantes com média de idade superior a 70 anos, reforça nossos achados.^35^ Neste trabalho, as atividades físicas foram categorizadas de acordo com a intensidade e mostraram uma redução em 40% na mortalidade para mulheres e 22% para homens que praticavam atividade física vigorosa.^36^

Por último, em nosso trabalho, o aumento da PAS mostrou-se como fator de proteção para mortalidade geral. Assim, para o aumento de 1 mmHg na PAS houve uma redução de 2% (IC 95% 1% a 3%) no risco de morte. Estes achados estão de acordo com os resultados da maioria dos estudos que identificaram uma relação inversa entre a pressão arterial e o risco de morte por causa cardiovascular ou qualquer outra causa, em pessoas com 80 anos de idade ou mais.^37^^,^^38^ No entanto, este assunto continua sendo gerador de discussões e proposições no meio científico. Os resultados conflitantes dos estudos de coorte e de alguns ensaios clínicos^39^ como os resultados do *Hypertension in the Very Elderly Trial* (HYVET) são difíceis, mas têm uma explicação plausível. No HYVET,^40^ foram incluídos participantes com pelo menos 160 mmHg e a meta para a PAS foi atingir níveis inferiores a 150 mmHg. Contrapondo com nosso estudo, que é de base comunitária e, portanto, não teve restrição de nível de PA, o risco dos indivíduos com PA muito baixa, provavelmente, superou o risco daqueles com PA elevada, o que poderia explicar nossos dados de proteção.

## Considerações e Limitações

Algumas limitações deste estudo devem ser consideradas, sendo o pequeno tamanho amostral a principal delas. A validação externa é limitada, uma vez que a população do estudo é uma fração de uma coorte muito específica, descendentes de italianos em um único local, portanto, não representativa da população idosa brasileira. Outra limitação que pode ser considerada é a inclusão de idosos enfermos, ou seja, com restrições físicas no mesmo grupo de idosos com gasto energético semanal inferior a 4.000 kcal. Estes idosos foram considerados sedentários, apesar desta ser uma situação do momento da avaliação e de não refletir exatamente seu hábito de vida. Contudo, somente 5% da amostra encontrava-se enferma e sem possibilidades de realizar qualquer atividade física.

O aspecto mais original do nosso estudo é a população investigada, idosos com 80 anos ou mais, um grupo não incluído, frequentemente, em estudos observacionais e ensaios clínicos. Adicionalmente, informações sobre a relação entre os genótipos da APOE/fatores de risco cardiovasculares clássicos e a mortalidade nessa faixa etária, especialmente no Brasil, são escassas. Os resultados do nosso estudo acrescentam uma contribuição relevante, tanto para a prevenção quanto para o manejo, dos fatores de risco nesta população.

## Conclusões

Considerando que a população está envelhecendo e o impacto dos fatores de risco tradicionais sobre os resultados pode não ser o mesmo que nas idades mais jovens, nossos resultados acrescentam uma contribuição relevante para a discussão sobre como controlar melhor os fatores de risco nessa população. Nosso estudo não evidenciou que idosos longevos portadores do alelo ε4 da APOE tivessem maior risco de morte que os portadores do genótipo de referência E3E3. De outra forma, a exposição a alguns fatores de risco relacionou-se de forma significativa à mortalidade geral na idade avançada, notavelmente, o tabagismo e o DM. Entretanto, a atividade física vigorosa e a PAS maior foram considerados como fatores de proteção.
