# Supplementary material for: Associação de Fatores de Risco Cardiovascular e Polimorfismo de APOE com Mortalidade em Idosos Longevos: Uma Coorte de 21 Anos
Source: Arq Bras Cardiol. 2020 Nov 1;115(5):873–81. [Article in Portuguese] doi: 10.36660/abc.20190263 (PMC8452209; doi:10.36660/abc.20190263)
Supplement: Translation

# Translation: Association of Cardiovascular Risk Factors and APOE Polymorphism with Mortality in the Oldest Old: A 21-Year Cohort Study

**Authors:** Lilian Vivian, Neide Maria Bruscato, Berenice Maria Werle, Waldemar de Carli, Renata Alonso Gadi Soares, Paulo Caleb de Lima Santos, Emilio Hideyuki Moriguchi

**Affiliations:** 1 VeranópolisRS Brazil Hospital Comunitário São Peregrino Lazziozi, Associação Veranense de Assistência em Saúde (AVAES), Veranópolis, RS - Brazil; 2 São PauloSP Brazil Laboratório de Genética e Cardiologia Molecular, Instituto do Coração (InCor), Hospital das Clínicas da Universidade de São Paulo (HC/FMUSP), São Paulo, SP - Brazil; 3 São PauloSP Brazil Universidade Federal de São Paulo Escola Paulista de Medicina - Departamento de Farmacologia, São Paulo, SP - Brazil; 4 Porto AlegreRS Brazil Universidade Federal do Rio Grande do Sul - Medicina Interna, Porto Alegre, RS- Brazil

**Keywords:** Cardiovascular Diseases, Risk Factors, Mortality, Apoliprotein E4, Aged, 80 and over

## Abstract

**Background:**

Knowledge of environmental and genetic factors for healthy aging in elderly people is controversial. In addition to this evidence, few studies have been designed for this population.

**Objectives:**

To investigate the relationship between the most frequent apolipoprotein E (APOE) genotypes and mortality in very elderly individuals living in a community and to evaluate survival according to cardiovascular risk factors.

**Methods:**

A sample of 74 elderly individuals aged ≥ 80 years, from the Veranópolis Project cohort, was selected for APOE genotyping. At baseline, anthropometric variables, glucose and lipid levels, blood pressure, and lifestyle variables (smoking, alcohol consumption, and physical activity) were collected. The Bayer Activities of Daily Living Scale was applied to their caregivers. Total study follow-up was 21 years. Two-sided p < 0.05 was considered statistically significant.

**Results:**

There was no association between APOE genotypes and mortality. However, the risk of death in elderly smokers was 2.30 times higher (hazard ratio [HR], 95% CI 1.01 to 5.24); in individuals with diabetes, it was 3.95 times higher (HR, 95% CI 1.27 to 12.30) than in individuals without diabetes. Subjects who practiced vigorous physical activity had a 51% reduction in risk of death (HR = 0.49, 95% CI 0.27 to 0.88). For an increase of 1 mmHg in systolic blood pressure, there was a 2% reduction (HR = 0.98, 95% CI 0.97 to 0.99) in risk of death.

**Conclusion:**

In this sample population, APOE genotypes were not associated with mortality. However, classic cardiovascular risk factors may be important for overall mortality in the very elderly.

## Introduction

The rapid growth in the elderly population worldwide has brought an increased interest in and need for studies on factors related to longevity with quality of life. Mortality data about elderly individuals aged 80 and over show that cardiovascular diseases (CVD) represent half of the causes of death.^1^ Despite the high frequency of chronic diseases, such as CVD and dementia, in this age range, they are usually excluded from well controlled studies or only analyzed as subgroups. Results from studies in the very elderly (aged 80 and over) are different from those in the young elderly (aged 60 to 74), for instance, higher mortality associated with reduction in diastolic pressure^2^ or systolic pressure^3^^,^^4^ and reduction in cholesterol,^5^ or a protective effect related to body mass index (BMI) above 30 kg/m^2^.^6^ However, other risk factors such as smoking^7^ and diabetes mellitus (DM)^8^ have been similarly associated, even at more advanced ages. Otherwise, a widely studied genetic factor, apolipoprotein E (APOE) polymorphism, more specifically the ε4 allele, appears as a risk factor for Alzheimer's disease (AD) in adults and young elderly.^9^^,^^10^ However, results from a specific cohort with very elderly identified a paradoxical effect of APOE ε2 allele associated with an increase in AD through post-mortem neuropathological criteria.^11^ Meta-analysis studies have shown that carriers of the APOE ε4 allele present a higher risk of early CVD.^12^^,^^13^ However, there are no studies to indicate whether this association is maintained in the oldest old. From this perspective, the objective of this study was to investigate the relationship between the most frequent APOE genotypes and mortality in very elderly individuals and to describe survival according to genotypes and exposure to classical cardiovascular risk factors.

## Methods

### Design

Prospective cohort study

### Study Population

The Veranópolis Project cohort started in 1994 following two comprehensive eligibility criteria: (1) age equal to or older than 80 years and (2) residing within the territorial domain of the municipality of Veranópolis, Rio Grande do Sul, Brazil. Recruitment of eligible individuals took place in 1994, 1996, and 1998. Summarily, the first recruitment happened during the month of July 1994 through an informal invitation by the research coordinator to participants in a religious service. Those present registered spontaneously and informed their intention to participate. During three weeks in July 1994, the researchers visited 100 elderly individuals at their residences or in community centers. In 1996, recruitment occurred through broadcasting in a local radio station; another 129 consented to take part in the study, and those who had participated in 1994 were reassessed. In 1998, a simple random sample of the participants in the previous years and another 13 new volunteers underwent APOE genotyping, and the main tests from the previous assessments. Thus, the Veranópolis Project cohort comprised 242 individuals, representing 87.4% of elderly individuals aged 80 and over who resided in the municipality between 1994 and 1998.^14^

During 2011 and 2012, vital status of the elderly individuals sampled for APOE genotyping (74 volunteers) was checked once more through home visits and, at that time, the Bayer Activities of Daily Living Scale (B-ADL) questionnaire was applied to their caregivers.

In December 2012, 18 years after the start of the cohort, 11 of the 242 members of were still living. The results presented in this manuscript refer to a period after verification of vital status from 2011 to 2012, that is, after the occurrence of the mortality outcome in all participants of the cohort of the oldest old, which occurred in 2015.

This study received approval from the Research Ethics Committee of the Federal University of Rio Grande do Sul, Brazil. All participants and/or their relatives signed an informed consent form.

### Variables

The APOE genotypes (rs7412 and rs429358) were used as a predictor variable, and the genetic factor was analyzed in two different periods during follow-up, the first in 1998^15^ and the second in 2011, including all living individuals who had not been sampled in 1998, namely, another 9 elderly individuals (methodology described in Alvim et al.^16^ (See Flowchart in Annex)

The outcome defined in this study was mortality from chronic CVD included in codes I00-I99 or from dementia included in codes F00-F03 of the International Classification of Diseases, 10th revision. To define cause of death in elderly individuals, copies of their death certificates were presented to two medical professionals, one geriatrician and one cardiologist, who were blinded to one another's assessments and to the genotypes of the deceased. In case of any divergence between the professionals concerning the cause of death, assessment was requested from a third professional. The final diagnosis, in case of an impasse, was defined by consensus between the three professionals. When cause of death could not be defined by the document alone, medical records were surveyed, and interviews were carried out with the family doctors or next of kin of the deceased.

The Veranópolis Project cohort is a broad study which seeks answers for the peculiar longevity of this population. Among the variables investigated in the cohort for this study, we selected those that are described as classical risk factors for CVD and those that could be independently associated with the outcome studied, namely, arterial hypertension, obesity, DM, dyslipidemia, smoking, alcohol abuse, and physical inactivity. Data from these variables were collected at the baseline of the year of inclusion of the elderly individual in the cohort (1994, 1996, or 1998). The baseline data collected in 1994 were re-evaluated in 1996. In 1998, data collection was repeated from a random sample from 1994 to 1996 and 13 additional individuals included in the cohort. For this study's data analysis, we used the information collected in the year of entry of the elderly individual in the cohort.

The methods used to measure cardiovascular risk factors and the justifications for the categorization, when applicable, are described succinctly below. Blood pressure (BP) was obtained using a mercury sphygmomanometer (Erka, Germany). Two or three measurements were taken according to variability, following the measurement intervals recommended by the guidelines, and the weighted average was calculated. To analyze data, BP was used as a quantitative, categorized variable, and individuals were considered hypertensive when BP ≥ 140/90 mmHg or when they were taking antihypertensive medication.^17^ Furthermore, pulse pressure, the result of subtracting diastolic blood pressure (DBP) from systolic blood pressure (SBP), was evaluated.

Obesity was defined by BMI; weight was measured with participants dressed lightly, without shoes, using mechanical scales (Filizolla, São Paulo). Height was determined standing upright, without shoes, using a measuring tape with shoulders in a normal position. To analyze the data, BMI was used as a continuous, categorical variable, with obesity^18^ and overweight^19^ being defined by the cut off points ≥ 30 kg/m^2^ (World Health Organization [WHO] and > 27 kg/m^2^ (Lipschitz), respectively.

For glycemia and lipid profile biochemical evaluations, venous blood samples were collected after 12 hours of fasting. A blood sampling system with disposable vacuum device (Vacutainer) in tubes with no anticoagulant was used. Plasmatic dosages were obtained through the manual technique of colorimetric enzymatic reaction with calibration standards and samples in duplicate. DM was defined as fasting glycemia ≥ 126 mg/dL or use of hypoglycemia medication.^20^ Dyslipidemia was evaluated through plasmatic dosages of triglycerides (TG), total cholesterol (TC), high-density lipoprotein cholesterol (HDL-C), and low-density lipoprotein cholesterol (LDL-C) calculation, which were used as quantitative, categorized variables according to the following criteria from the V Brazilian Guidelines on Dyslipidemias and Prevention of Atherosclerosis^21^ and the American Association of Clinical Endocrinologists' Guidelines for Management of Dyslipidemia and Prevention of Atherosclerosis,^22^ TG ≥ 150 mg/dL; TC ≥ 200 mg/dL; HDL-C < 50 mg/dL for women and < 40 mg/dL for men; LDL-C ≥ 160 mg/dL. LDL-C was obtained using Friedewald's formula for TG values below 400 mg/dL.

The lifestyle variables smoking, alcohol abuse, and physical inactivity were obtained using a standardized questionnaire applied at baseline. Smoking was evaluated through statements on consumption or non-consumption of tobacco (cigarette, corn straw cigarette, pipe). Two groups were considered: 1) non-smokers: individuals who never smoked; 2) smokers or ex-smokers. Alcohol abuse was evaluated through statements on the amount of alcohol consumed per week, and abuse was considered at values of > 210 g/week for men and > 105 g/week for women.^23^ Physical inactivity was evaluated through statements on daily activities during a normal week of work and leisure. Two different cut-off points were used, < 2,000 kcal/week^24^^,^^25^ and < 4,000 kcal/week,^26^ which are the minimum amounts of physical activity to attain major cardiovascular benefits such as attenuated thickening of the inner-middle layer of carotid arteries,^24^ increase in HDL-C^25^ and reduction of mortality in patients with coronary arterial disease.^26^ The instrument used to report different physical activities comprised a list of 27 habitual activities in the routine of people who live in the city and in the country, and one further open question about any other activity besides the pre-selected ones. Participants in the study were asked to report time spent in minutes and the weekly frequency practicing these activities. Therefore, following the same rationale for other studies on the very elderly, we chose to use energy expenditure calculated in kilocalories per week (kcal/week), considering participants' weight, the reported time duration of the activity, the metabolic equivalents (MET) for the specific activity^27^ and the weekly frequency of the activities: Energy expenditure (kcal/week) = MET X weight (kg) X time duration of activity (minutes) / 60 X weekly frequency. We thus deem that this measurement better reflects the very elderly energy expenditure in the community studied (rural and urban) than the simply measurement of MET that is usually described in current works.

B-ADL was applied by a researcher, who was trained and blinded in relation to participants' genotypes, to their caregivers in the period from August 2011 to December 2012. The instrument was employed as a way of identifying cases of dementia and the result used as a potentially confounding variable, as it has been well described that the APOE ε4 allele is a risk factor for the development of AD.^9^^,^^10^ The score obtained from the B-ADL ranges from 1.00 to 10.00, and higher scores represent greater difficulty in the activities. To analyze the data, the B-ADL score was used as a quantitative, categorized variable, using a cut-off point of ≥ 3.12 to define cases of dementia.^28^

### Statistical Analysis

Quantitative variables were described as average and standard deviation or median and interquartile amplitude. For comparison between groups, we used Student's t test for independent (unpaired) samples (Shapiro-Wilk normality test) and, in case of asymmetry, the Mann-Whitney test. Qualitative variables were described as absolute and relative frequencies. For comparing proportions between groups, the Pearson chi-square test or Fisher exact test were applied. We used the the Kaplan-Meier survival curve estimate method to evaluate survival time and the log-rank chi-square test for comparison between groups.

To control confounding factors in relation to death, Cox proportional hazards model was used. As a measure of effect, the hazard ratio (HR) was calculated, with respective 95% confidence intervals (CI). The criterion for entering a variable in the multivariate model was that it presented p value < 0.20 in univariate analysis.

The level of significance was p < 0.05, and the data were analyzed with Statistical Package for the Social Sciences software version 21.0.

## Results

In this study, the sample of 74 individuals of the Veranópolis cohort had a median follow-up time of 9 years (P25 – P75: 6 – 14 years), ranging from 0.6 to 21 years. It is worth underscoring that there were no follow-up losses in this sample. Based on statements by the elderly individuals, 94.6% descended from Italian immigrants. The APOE gene (allele) frequency present in the sample was 4.1% ε2; 85.1% ε3, and 10.8% ε4. The genotype frequency was 1.4% E2E2; 5.4% E2E3; 71.6% E3E3, and 21.6% E3E4. The genotype distribution is in Hardy-Weinberg equilibrium (χ^2^ = 0.07; degree of freedom = 1; p = 0.79). No carriers of the E2E4 and E4E4 genotypes were found in the sample. Therefore, only the E3E4 formed the exposed group, that is, carriers of the APOE ε4 risk allele. Table 1 summarizes the characteristics of the groups of interests. The complete table, including all the described variables, can be found in a quantitative, categorized form in Additional File 1: Complete Table 1.

**Table 1:** Characterization of the sample at baseline

Variables	Total sample	E3E3 genotype	E3E4 genotype	p*
	(n = 69)	(n = 53)	(n = 16)	
Age at entry (years)	82.6 2.8	82.2 2.7	84.0 ± 2.8	0.021
Sex				0.267
Male	23 (33.3)	20 (37.7)	3 (18.8)	
Female	46 (66.7)	33 (62.3)	13 (81.3)	
B-ADL†	2.98 (1.44-5.55)	2.95 (1.38-5.46)	3.19 (1.54-7.44)	0.631
Smoking				0.717
Non-smoker	56 (81.2)	42 (79.2)	14 (87.5)	
Smoker/ex-smoker	13 (18.8)	11 (20.8)	2 (12.5)	
Alcohol abuse				0.093
No	17 (24.6)	13 (24.5)	4 (25.0)	
Yes	18 (26.1)	17 (32.1)	1 (6.3)	
Teetotalers	34 (49.3)	23 (43.4)	11 (68.8)	
BMI (kg/m2)†	26.8 ± 5.4	27.6 ± 5.5	23.7 ± 3.6	0.011
Physical activity (kcal/week)	5133 (2386-9846)	5421 (2155-10544)	3580 (2300-6930)	0.216
SBP (mmHg)	161 ± 25.3	162 ± 23.7	158 ± 30.5	0.489
DBP (mmHg)	86.3 ± 13.2	88.6 ± 12.7	78.5 ± 12.4	0.007
Pulse pressure (SBP-DBP)	75.1 ± 22.7	73.9 ± 21.5	79.0 ± 26.8	0.439
Hypertension	62 (89.9)	48 (90.6)	14 (87.5)	0.660
Fasting glycemia (mg/dL)‡	95.4 ± 20.7	96.2 ± 22.7	92.6 ± 12.2	0.539
Total cholesterol (mg/dL)‡	209 ± 48.8	203 ± 43.9	229 ± 59.5	0.066
LDL cholesterol (mg/dL)§	138 ± 42.1	132 ± 36.0	156 ± 55.0	0.055
HDL cholesterol (mg/dL)‡	43.4 ± 11.4	44.0 ± 12.5	41.4 ± 6.6	0.284
Triglycerides (mg/dL)‡	102 (85.8-146)	102 (83.4-144)	107 (88.2-159)	0.806

Results described as average ± standard deviation, median (25 – 75 percentiles) or n (%).

* t-student test (comparison of averages), Mann-Whitney test (comparison of medians), Pearson chi-square test (categorical variables) or Fisher exact test (for the variables of smoking and hypertension);

† variables analyzed in 15 individuals with the E3E4 genotype;

‡ variables analyzed in 52 individuals with the E3E3 genotype;

§ variable analyzed in 51 individuals with the E3E3 genotype. B-ADL: The Bayer Activities of Daily Living Scale; BMI: body mass index; SBP: systolic blood pressure; DBP: diastolic blood pressure; LDL: low density lipoprotein; HDL: high density lipoprotein.

The causes of death between the E3E3 and E3E4 groups are summarized in Table 2. We point out that average life expectancy for individuals was 92.3 years (95% CI 91.2 to 93.4). For comparison, the table presents the levels of significance without adjustment and adjusted for variables with p < 0.2 in univariate analysis.

**Table 2:** Comparison of outcomes between genotypes

Outcomes	Sample total (n = 69)	Genotype E3E3 (n = 53)	Genotype E3E4 (n = 16)	p	p^adjusted^†
	n (%)	n (%)	n (%)		
Death	69 (100)	53 (100)	16 (100)	–	–
Cause of death				0.216*	0.302
Cardiovascular	43 (62.3)	36 (67.9)	7 (43.8)		
Dementia	6 (8.7)	4 (7.5)	2 (12.5)		
Other	20 (29.0)	13 (24.5)	7 (43.8)		

* Pearson chi-square test;

† adjusted for age of entry in the study, alcohol consumption, body mass index, physical activity ≥ 4,000 kcal/week, diastolic blood pressure, and total and low-density lipoprotein cholesterol

To evaluate survival of the elderly according to the APOE genotypes, the Kaplan-Meier survival curve estimate method was used, represented in the graph in Figure 1. We observed that there was no association between APOE polymorphisms and survival (logrank = 0.955) in the very elderly in this sample.

**Figure 1:** (A) Kaplan-Meier survival curve for carriers of the E3E3 and E3E4 genotypes. (B) Survival probability for the groups with a periodicity of 2 years.

Additionally, it is pertinent to analyze cardiovascular risk factors associated with the mortality in very elderly, since this age range usually presents singular and, at the same time, contradictory results. In order to do this, Kaplan-Meier survival estimates were used with Cox regression to control confounding variables. The results are presented in Table 3 (and in Additional File 2: Complete Table 3). The survival curves for the categorical factors associated with death can be viewed in Figure 2.

**Table 3:** Univariate and multivariate Cox regression analysis for factors associated with mortality

Variables	Univariate	p	Multivariate*	p
	HR (95% CI)		HR (95% CI)	
Age (years)†	1.19 (1.10 – 1.30)	< 0.001	1.24 (1.12 – 1.39)	< 0.001
Males	1.45 (0.88– 2.39)	0.150	1.04 (0.47 – 2.32)	0.920
E3E4 genotype	1.35 (0.77 – 2.39)	0.299	–	–
B-ADL	0.93 (0.85 – 1.02)	0.119	0.92 (0,82 – 1.02)	0.102
Smoker/ex-smoker	2.37 (1.29 – 4.36)	0.005	2.30 (1.01 – 5.24)	0.047
Alcohol consumption (g/week)	1.00 (0.99 – 1.00)	0.602	–	–
BMI	0.94 (0.90 – 0.99)	0.018	0.96 (0.91 – 1.01)	0.107
Physical activity ≥ 4,000 kcal/week	0.55 (0.34 – 0.89)	0.016	0.49 (0.27 – 0.88)	0.017
SBP	0.99 (0.98 – 1.00)	0.050	0.98 (0.97 – 0.99)	0.018
DBP	0.97 (0.94 – 0.99)	0.016	1.01 (0.98 – 1.04)	0.669
Pulse pressure	0.99 (0.98 – 1.01)	0.392	–	–
Hypertension	0.53 (0.24 – 1.19)	0.123	1.35 (0.54 – 3.38)	0.516
Diabetes mellitus	5.10 (1.70 – 15.3)	0.004	3.95 (1.27 – 12.3)	0.018
Total cholesterol ≥ 200 mg/dL	0.52 (0.32 – 0.86)	0.010	0.74 (0.42 – 1.31)	0.303
LDL ≥ 160 mg/dL	0.69 (0.41 – 1.18)	0.175	1.00 (0.99 – 1.00)	0.410
Low HDL‡	1.46 (0.86 – 2.47)	0.159	1.03 (0.57 – 1.84)	0.932
Triglycerides ≥ 150 mg/dL	1.23 (0.69 – 2.20)	0.489	–	–

* The criterion for entering the variable in the multivariate model was p value < 0.20 in univariate analysis.

† Age at the time of cohort recruitment (study entry).

‡ Low HDL refers to HDL-cholesterol < 40 mg/dL for men and < 50 mg/dL for women. HR: hazard ratio; 95% CI: 95% confidence interval; B-ADL: The Bayer Activities of Daily Living Scale; BMI: body mass index; SBP: systolic blood pressure; DBP: diastolic blood pressure; LDL: low density lipoprotein; HDL: high density lipoprotein.

**Figure 2:** Survival curves for factors associated with mortality in the very elderly: (A) smoking, (B) vigorous physical activity [PA] and (C) diabetes mellitus.

Considering that the sample for this study comprised elderly individuals selected at two moments of follow-up, there may have been a selection bias. Specifically, the nine elderly individuals that were genotyped in 2011 would form a group of survivors. With the intention of weighing this bias, we performed new analyses without these individuals. In this manner, we obtained very similar results, including in relation to the sample descriptive level, with the exceptions of smoking and diabetes, which lost the association with mortality. In this new analysis, the risk of death in smokers and ex-smokers was 2.14 (95% CI 0.93 to 4.91) in the multivariate model (p = 0.075).

## Discussion

### APOE Polymorphism

Review studies have shown that genetic frequencies related to APOE polymorphism are highly variable, especially in regard to the ε4 allele.^29^ The gene frequency observed in the present study is similar to that found in the population in Italy.^30^ Since the sample for this work comprised 94.6% Italian immigrant descendants, this similarity was expected, and it indicates that the sampling process was adequate.

Curiously, in our results, individuals with the E3E4 genotype presented an average age significantly older than E3E3 individuals (Table 1). We believe this difference to be casual, since relevant publications indicate that there is no difference in mortality between carriers of the E3E3 and E3E4 genotypes before 80 years of age.^31^^,^^32^ Among the risk factors for CVD investigated, BMI and DBP presented significant differences between the evaluated genotypes. In those cases, individuals in the E3E3 group presented an average BMI classified as overweight (WHO) or overweight (Lipschitz) and higher DBP. We did not find similar data to these in studies previously published on the community basis, but similar comparisons in larger samples in the adult to young elderly age range suggest that there is no association between obesity^33^ or DBP levels^34^ and the APOE genotypes.

### Classical Cardiovascular Risk Factors

Our results have indicated that some cardiovascular risk factors are associated with general mortality, even in the very elderly age group, in which the majority of these classical factors lose their predictive power for risk. Smoking appeared to be important in this relation. The risk of death in smokers and ex-smokers was 2.30 (95% CI 1.01-5.24) times that of non-smokers. A meta-analysis study^7^ brings evidence that smoking remains a strong risk factor for mortality in elderly individuals aged 80 and over as well. In relation to elderly individuals with diabetes, despite the low percentage (6%) present in our sample, this number was enough to attain a significant difference. Very elderly individuals with diabetes had 3.95 (95% CI 1.27-12.3) times higher risk of death in comparison with those without diabetes, showing that this risk factor remains important, even over 80 years of age. A similar result became evident in the very elderly cohort of The Adventist Health Study.^8^ Vigorous physical activity appeared as a protective factor against mortality in our study. Individuals who expended more than 4,000 Kcal/week through work and leisure activities had a 51% reduction in the risk of death (95% CI 12% to 73%). Regarding practice of vigorous activities, a study that combined two Australian cohorts, the Australian Longitudinal Study on Women's Health and the Health in Men Study, with more than 18,000 participants with average age over 70, reinforces our findings.^35^ In this study, physical activities were categorized according to intensity, and they showed a 40% reduction in mortality for women and a 22% reduction for men who practiced rigorous physical activity.^36^

Finally, in this study, the increase in SBP appeared as a protective factor against general mortality. Thus, an increase of 1 mmHg in SBP reduced the risk of death by 2% (95% CI 1-3%). These findings are in accordance with the results from most studies that identified an inverted reaction between BP and risk of death from cardiovascular or any other causes in persons aged 80 or older.^37^^,^^38^ However, this subject still generates discussions and propositions in the scientific community. The conflicting results from cohort studies and some clinical trials,^39^ such as the results from the Hypertension in the Very Elderly Trial (HYVET) are difficult, but there is a plausible explanation. The HYVET^40^ included participants with at least 160 mmHg, and the target for SBP was to attain levels below 150 mmHg. In comparison, our study, which is community based and therefore had no BP restrictions, the risk for individuals with very low BP probably surpassed the risk for those with high BP, which could explain our data showing protection.

### Considerations and Limitations

Some limitations in this study must be considered; the small sample size is the main one. External validation is limited, given that the study population is a fraction of a very specific cohort, namely, Italian descendants in a single location, and is not, therefore, representative of the Brazilian elderly population. Another shortcoming that could be considered was the inclusion of elderly patients who were sick, that is, with physical restrictions, in the same group of elderly individuals with weekly energy expenditure below 4,000 kcal. They were considered as sedentary, although this was the situation at the moment they were evaluated and it does not exactly reflect their lifestyle. Nevertheless, only 5% of the sample were sick and unable to practice any physical activity.

The most original aspect of our study is the study population, namely, elderly individuals aged 80 years or older, a group that is not frequently included in observational studies and clinical trials. Furthermore, information on the relationship between APOE genotypes/classic cardiovascular risk factors and mortality in this age group is lacking, especially in Brazil. The results of our study add a relevant contribution to both prevention and management of risk factors in this population.

## Conclusions

Considering that the population is getting older and the impact of traditional risk factors on outcomes may not be the same as it is in younger ages, our results add a relevant contribution to the discussion on how to better control risk factors in this population. Our study did not find evidence that very elderly carriers of the APOE ε4 allele were at greater risk of death than carriers of the E3E3 reference genotype. In contrast, exposure to some risk factors was significantly related to general mortality at an advanced age; namely, smoking and DM were characterized as risk factors. However, vigorous physical activity and higher SBP were protective factors.
